# Sun safety knowledge, practices and attitudes in rural Australian farmers: a cross-sectional study in Western New South Wales

**DOI:** 10.1186/s12889-021-10777-x

**Published:** 2021-04-15

**Authors:** Charmaine D’Souza, Nikitha Kramadhari, Elizabeth Skalkos, Tegan Dutton, Jannine Bailey

**Affiliations:** Bathurst Rural Clinical School, School of Medicine, Western Sydney University, PO Box 9008, Bathurst, NSW 2795 Australia

**Keywords:** Farmers, Sun protection, Skin Cancer, Melanoma, Occupational exposure

## Abstract

**Background:**

Rates of skin cancer in Australia are amongst the highest in the world, with Western New South Wales (NSW) exhibiting very high prevalence. There is a large proportion of outdoor workers, including farmers, in Western NSW who have high levels of sun exposure and hence are at greater risk of developing skin cancer.

**Aims:**

To characterise the current sun safety practices of farmers in Western NSW and explore their knowledge, attitudes and perceived barriers towards sun safety and its implementation.

**Methods:**

A cross-sectional survey study was conducted using a self-directed questionnaire. Participants were recruited at field days held in Western NSW and through surveys distributed at general practices, local stores and online. Eligibility criteria were aged 18 years and over and currently working on a farm in the study region**.**

**Results:**

Of the 144 participants, 89 (61.8%) were male with a mean age of 49 years. Knowledge of sun safety was relatively high with most questions answered correctly by greater than 80% of participants. Risk of developing skin cancer was underestimated in 58 (40.3%) participants. Of all participants, 89 (62.2%) identified one or more barriers to practicing sun safety. The most common barrier was forgetfulness in 62 (43.4%) participants. The identification of barriers was significantly associated with reduced engagement of sun safety practices (*p* = 0.009).

**Conclusions:**

Knowledge of sun safety among farmers was high. There was, however, underestimation of risk of developing skin cancer. Addressing perceived barriers to implementing sun safety could improve sun safety practices in this cohort.

## Background

Rates of skin cancer in Australia are some of the highest in the world and are rising [1]. New South Wales (NSW) is the state with the highest incidence of deaths caused by melanoma skin cancer, and Western NSW has one of the highest mortality rates related to melanoma in Australia (1.2 times the national average) [2, 3]. In Australia, however, 97% of all skin cancers are non-melanoma skin cancers [4]. Recent data projections reported that 4 Australians per day lose their lives due to non-melanoma skin cancer and that the cost to the Australian healthcare system is $1.2 billion [4]. The burden on those residing in rural areas is significantly higher than that of their urban counterparts [4]. Further, Australian farmers have a mortality rate from skin cancer that is 60% higher than the general population [5].

The most important modifiable risk factor for developing skin cancer is exposure to ultraviolet radiation (UVR) [6, 7], both naturally occurring (e.g. solar UVR) and artificial (e.g. UVR emitting tanning devices). Outdoor workers, including farmers, who are exposed to large amounts of natural UVR are at high risk for developing skin cancer [8] and the development of eye problems such as cataracts and macular degeneration, as a result of their high levels of occupational UVR exposure [9, 10]. One systematic review found Australian farmers had on average 6–8 times more UVR exposure than their indoor counterparts [11].

Since the 1980s, numerous public health campaigns have attempted to educate and encourage the use of sun protection, which has been demonstrated to reduce the risk of skin cancer [12]. The most well-known of these is “Slip! Slop! Slap!,” which refers to the use of protective clothing, sunscreen and a hat respectively [12]. In 2005, ‘Seek’ shade, and ‘Slide’ on sunglasses were added to the campaign [13].

There have been relatively few studies on the use of sun protection in farmers and their knowledge of and attitudes towards it. This is an important research gap that needs to be filled given the predicted future impacts of ozone depletion and climate change on the outdoor work environment [14]. Most of the current research has involved small sample sizes and/or been performed on single communities or at single events [15–19]. A study of American farmers reported that hats with wide brims or back flaps, shirts with long-sleeves or collars, sunscreen with a Sun Protection Factor (SPF) of ≥15 or sunglasses were used 50% or less of the time [15]. Another study from North Carolina found that while farmworkers frequently wore long-sleeved shirts and baseball caps or visors, they rarely used wide brim hats, sunscreen or sunglasses [16]. These results were consistent with a review of the literature on sun safety in farmers conducted by Kearney et al. [17] and an Italian study conducted with outdoor workers [18]. Evidence showed that females were more likely than males to practice sun protection behaviours including wearing wide brim hats, sunglasses, long-sleeve shirts, using sunscreen and seeking shade [17, 19].

A qualitative study of Bavarian farmers explored knowledge, attitudes and barriers related to sun safety [20]. Half the participants thought they were at increased risk of non-melanoma skin cancer compared to the general population. Further, older farmers have been found to assign higher risk to sun exposure than younger farmers [17, 20]. Women compared to men have also displayed more interest in sun safety [20]. Inconvenience and discomfort are common perceived barriers, especially related to using sunscreen.

The literature to date has largely focussed on overseas populations and may have reduced relevance to Australian farmers. One Victorian study on sun safety beliefs and practices of Australian farmers reported that while 73% of farmers believed they were at risk of developing skin cancer, only 35% wore a wide brim hat, 27% wore long sleeved shirts, 48% never wore sunscreen on all exposed skin and 59% did not have sunscreen available when working outdoors; 75% of farmers, however, wore long trousers [21]. Similar findings were reported in a study conducted in the New England region of NSW, where the most frequently reported sun-protective behaviours were wearing a shirt with a collar (44.8%) followed by wearing a wide brimmed hat (33.9%) and using sunscreen was reported in just 11% of the population [19]. Neither of these studies explored sun safety knowledge levels in farmers.

To our knowledge, there is currently no literature on practices, knowledge, attitudes or barriers towards sun safety in farmers in the Western NSW region. The primary aim of this study was to characterise the current sun safety practices of farmers in Western NSW. Secondary aims were to explore their knowledge of, attitudes towards, and perceived barriers to implementing sun safety on the farm.

## Methods

A cross-sectional survey study was conducted. Ethics approval was granted by the Western Sydney University Human Research Ethics Committee (H11327).

### Data collection instrument

Data was collected using a self-directed, anonymous survey with multiple choice questions, Likert scales and some short answer questions. The survey was adapted from two previously published surveys and related literature on the topic [13, 15, 21]. This is because there are currently no gold standard validated instruments for sun safety practices. The survey covered participant demographics, skin type using the Fitzpatrick classification scale, self-reported sun safety practices, skin cancer history, sun safety knowledge, attitudes and perceptions, and barriers to implementing sun safety on the farm [8, 15, 22].

### Participants and recruitment

To be included in this study, participants needed to be: aged 18 years or above, currently working in Western NSW and engaging in farm activities through employment or on one’s own personal farm. Western NSW was defined as the region encompassed by the Western NSW Primary Health Network [23]. Participant postcode was used to confirm this eligibility criterion. There were no specific exclusion criteria for the study. A convenience sample of participants was recruited from July to November 2019 at public events including farming field days, farmer’s markets and agricultural shows and completed a hard copy of the survey. Relevant farming organisations and Facebook groups within the WNSW PHN were contacted via telephone, email and Facebook messaging to distribute an online link to the online version of the survey which was hosted on Qualtrics (https://www.qualtrics.com/).

### Data analysis

Hard copy survey data were uploaded to the online survey platform after which all data were downloaded as a Microsoft Excel spreadsheet for data analysis in SPSS (IBM SPSS Statistics, version 25; https://www.ibm.com/au-en/analytics/spss-statistics-software). Data were described using summary statistics. For categorical data, chi square tests, or Fisher’s exact test where appropriate, were used to determine the association between relevant demographic factors and sun protection practices, knowledge and attitudes. Sun safety practice items were summed to create a sun safety practice score for each individual participant with a maximum possible score being 29 and minimum being 0. A knowledge score was similarly generated from responses to the knowledge questions, with a minimum score of 0 and maximum of 10. For continuous data, normality of the data was confirmed using Kolmogorov-Smirnov tests, and independent t-tests and one-way ANOVAs (with post hoc Tukey tests) were included in the analysis. Pearson correlations were used to explore for correlations between continuous data variables. Missing responses were excluded from the analyses. Statistical significance was set at *p* < 0.05.

## Results

There were 157 responses in total, of which 13 were excluded because they did not meet the inclusion criteria, leaving 144 responses for analysis. Of all the participants, 89 (62.2%) were male and 54 (37.8%) were female (Table 1). The age range was 18–89 years with a mean of 49 ± 18 years. Participants were relatively evenly distributed across education levels, with an education level up to Year 10 (29.4%, *n* = 43) most common. The demographic profile is relatively consistent with that reported for Australia’s farming workforce in that 32% are female and the median age of the workforce is 49 years [24]. Roles on the farm were classified into farm owner (25.2%, *n* = 36), farm manager (39.2%, *n* = 56), farm worker (28.7%, *n* = 41) and other (7.0%, *n* = 10). The primary agricultural commodities included cattle, cropping, livestock, sheep and other. Most participants reported having Type 2 (30.7%, *n* = 43) skin, which usually burns, or Type 3 (45.7%, *n* = 64) skin which sometimes burns, according to the Fitzpatrick scale.

**Table 1:** Participant demographics

Demographic	n (%), unless otherwise stated
**Age** (years), mean ± SD (range)	49 ± 18 (18–89)
**Gender**, *n* = 143	
Male	89 (62.2)
Female	54 (37.8)
**Role on farm**, *n* = 143	
Worker	41 (28.7)
Manager	56 (39.2)
Owner	36 (25.2)
Other	10 (7.0)
**Primary agricultural commodity**, *n* = 136^a^	
Cattle	52 (26.9)
Cropping	32 (16.6)
Livestock	8 (4.1)
Sheep	73 (37.8)
Other (apples, honey, pigs, horses, timber, vegetables)	16 (8.2)
Not specified	12 (6.2)
**Highest education level**, *n* = 143	
Primary or secondary school (year 10 or prior)	43 (30.1)
Secondary school (year 12)	25 (17.5)
Diploma/certificate qualification	42 (29.4)
Degree qualification or post-graduate qualification	33 (23.1)
**Annual household income**, *n* = 130	
Less than $50,000	38 (29.2)
$50,000 - $75,000	32 (24.6)
$76,000 - $100,000	28 (21.5)
More than $100,000	32 (24.6)
**Hours spent in the sun daily**, *n* = 141	
Less than 0.5 h	3 (2.1)
0.5–2 h	10 (7.1)
2–4 h	23 (16.3)
4–6 h	41 (29.1)
6–8 h	23 (16.3)
More than 8 h	41 (29.1)
**Number of red or painful sunburns in the last year**, *n* = 140	
0	53 (37.9)
1	38 (27.1)
2	22 (15.7)
3	16 (11.4)
4	5 (3.6)
≥ 5	6 (4.3)
**Fitzpatrick scale skin type**, *n* = 140	
Type 1	13 (9.3)
Type 2	43 (30.7)
Type 3	64 (45.7)
Type 4	17 (12.1)
Type 5	3 (2.1)
Type 6	0 (0)
**Have ever examined themselves for skin cancer**, *n* = 142	
Yes	103 (72.5)
No	39 (27.5)
**Have ever seen a health professional for a skin check**, *n* = 143	
Yes	113 (79.0)
No	30 (21.0)
**Have ever had a skin cancer removed**, *n* = 143	
Yes	60 (42.0)
No	83 (58.0)
**A sun safety protocol is in place at the farm**, *n* = 138	
Yes	63 (45.7)
No	69 (50.0)
Not sure	6 (4.3)
**Report the presence of a sun safety protocol by farm role type,** ***n*** **= 137**	
Farm owner	16 (25.8)
Farm manager	24 (38.7)
Farm worker	18 (29.0)
Other	4 (6.5)

^a^ Some participants named more than one commodity

Most participants (72.5%, *n* = 103) had examined themselves for skin cancer at least once during their lifetime (Table 1) and most had seen a health professional for a skin check at some point (79.0%, *n* = 113). There was a fairly even divide between workplaces that did and did not have a sun safety protocol in place (45.7 and 50.0%, respectively). Reporting of a sun safety protocol was not associated with role type (*p* = 0.261).

When asked whether they have ever had a skin cancer removed, basal cell carcinoma (BCC, 22.9%, *n* = 24) and sunspot/actinic keratosis (22.9%, *n* = 24) were the most commonly reported. These were followed by squamous cell carcinoma (9.5%, *n* = 10) and melanoma (4.8%, *n* = 5). Some participants were not sure about the type of skin cancer they had had removed (14.3%, *n* = 15).

The sun safety practice scores stratified by demographic variables are presented in Table 2. A higher score indicates greater use of sun safety practices. The presence of barriers to implementing sun safety was significantly associated with sun safety practice score (*p* = 0.024), with those who reported one or more barriers being present (mean 13.9, SD 4.2) having a lower sun safety practice score than those who reported no barriers (mean 15.5, SD 4.0). Sun safety practices did not differ significantly between those who have previously had a skin cancer removed and those who have not (*p* = 0.644). Those with a skin type less likely to burn (Fitzpatrick scale type 4 or 5) showed a trend towards fewer sun safety practices compared to those with type 1 or 2 skin, however this did not reach statistical significance. No other demographic variables showed significant associations with sun safety practice scores.

**Table 2:** Sun safety practice score in farmers by characteristics

Characteristic	n	Mean (SD)	***t***-score	***F***-value	***P***-value
**Age group (years)**, *n* = 141			–	0.728	0.537
< 25	12	15.9 (4.2)			
25–44	41	14.0 (3.5)			
45–64	58	14.3 (4.0)			
≥ 65	30	14.8 (5.3)			
**Gender**, *n* = 143			−0.477	–	0.634
Male	89	14.4 (4.2)			
Female	54	14.7 (4.2)			
**Highest education level**, *n* = 143			–	0.759	0.581
Primary school	2	12.0 (11.3)			
Secondary school (Year 10 or prior)	41	14.8 (4.5)			
Secondary school (Year 12)	24	14.4 (3.8)			
Diploma or certificate	42	15.1 (3.9)			
Degree	25	13.6(3.9)			
Post-graduate qualification	9	13.4 (4.4)			
**Annual household income**, *n* = 129			–	1.132	0.339
Less than $50,000	37	14.7 (4.7)			
$50,000 - $75,000	32	14.9 (3.9)			
$76,000 - $100,000	28	13.6 (3.9)			
More than $100,000	32	13.5 (3.9)			
**Hours spent in the sun daily**, *n* = 140			–	0.828	0.532
Less than 0.5 h	3	15.0 (8.9)			
0.5–2 h	10	13.6 (5.7)			
2–4 h	22	14.5 (4.9)			
4–6 h	41	15.4 (3.5)			
6–8 h	23	13.3 (3.7)			
More than 8 h	41	14.7 (4.0)			
**Fitzpatrick scale skin type**, *n* = 140			–	1.338	0.259
Type 1	13	15.0 (4.7)			
Type 2	43	15.6 (4.6)			
Type 3	64	14.3 (3.8)			
Type 4	17	13.5 (4.2)			
Type 5	3	12.0 (1.0)			
**Number of red or painful sunburns in the last year**, *n* = 139			–	1.802	0.117
0	52	14.2 (4.7)			
1	38	15.7 (3.7)			
2	22	15.4 (2.4)			
3	16	12.3 (3.8)			
4	5	14.6 (6.9)			
≥ 5	6	14.5 (4.8)			
**Barriers to implementing sun safety**, *n* = 143			−2.274	–	**0.024**
Present	89	13.9 (4.2)			
Absent	54	15.5 (4.0)			
**Had a skin cancer removed**, *n* = 143			0.463	–	0.644
Yes	60	14.7 (4.2)			
No	83	14.4 (4.2)			

Sun safety practices, item by item, are presented in Table 3. About two-fifths of participants reported that they never use no sun protection (45.0%, *n* = 58). A further two-fifths (40.3%, *n* = 52) reported that they only sometimes forego sun protection. The most commonly engaged in sun safety practice was the wearing of sunglasses, followed by wearing long trousers and wearing a long sleeve shirt. Regarding type of hat, a wide brimmed hat was most commonly worn (69.6%, *n* = 96) and only four participants (2.9%) wore no hat.

**Table 3:** Sun safety practices item by item amongst farmers in western New South Wales

Sun safety practice	n (%)				
	Never	Sometimes	Half the time	Most of the time	Always
**Reapply sunscreen every 2 h**	41 (28.9)	56 (39.4)	19 (13.4)	19 (13.4)	7 (4.9)
**Wear sunglasses**	16 (11.3)	15 (10.6)	8 (5.6)	34 (23.9)	69 (48.6)
**Wear a long sleeve shirt**	8 (5.7)	24 (17.1)	18 (12.9)	36 (25.7)	54 (38.6)
**Wear long trousers**	4 (2.8)	17 (12.1)	28 (19.9)	36 (25.5)	56 (39.7)
**Use a portable shade**	19 (13.5)	49 (34.8)	25 (17.7)	28 (19.9)	20 (14.2)
**Avoid sun during peak times**	33 (23.6)	56 (40.0)	19 (13.6)	26 (18.6)	6 (4.3)
**Use no sun protection**	58 (45.0)	52 (40.3)	7 (5.4)	7 (5.4)	5 (3.9)
	**Wide brimmed**	**Bucket hat**	**Legionnaire’s**	**Cap**	**No hat**
**Which hat do you wear most often?**	96 (69.6)	9 (6.5)	2 (1.4)	27 (19.6)	4 (2.9)

Table 4 shows participant knowledge and attitudes about sun safety. On average, five out of ten sun safety knowledge statements were answered correctly by more than 90% of participants. About one third of participants (31.3%, *n* = 45) correctly identified that sun exposure helped to improve mood. Females were more likely to answer this question correctly than males (*p* = 0.032). The mean sun safety knowledge score for the cohort was 8.4 (SD 1.4).

**Table 4:** Knowledge and attitudes about sun safety amongst farmers in western New South Wales

**Knowledge questions**		**Answered correctly, n (%)**
Is prolonged exposure to sun harmful?		139 (96.5)
Sun exposure is healthy because:	Method of skin tanning	136 (94.4)
	Source of vitamin D exposure	117 (81.3)
	Helps to improve mood	45 (31.3)
	Not healthy at all	122 (84.7)
UV radiation can cause the following:	No health problems	139 (96.5)
	Skin cancer	138 (95.8)
	Premature aging	122 (84.7)
	Sunburn	133 (92.4)
	Cataracts	96 (66.7)
**Attitude Statements**		**Agreed with the statement, n(%)**
Using sun protection is important to me		126 (87.5)
Using sun protection is convenient for me		104 (72.2)
I think I am at higher risk of developing skin cancer compared to the average population		86 (59.7)

Of the 144 participants, 126 (87.5%) agreed that using sun protection is important to them, and 104 (72.2%) believed that using sun protection is convenient (Table 4) demonstrating positive attitudes towards sun safety on average. Additionally, 86 (59.7%) participants agreed that they are at higher risk of developing skin cancer compared to the average population. Chi square analyses found no statistically significant association between attitudes to sun safety and demographic variables, including whether they had previously had a skin cancer removed and skin cancer type (data not shown).

**Fig. 1:**
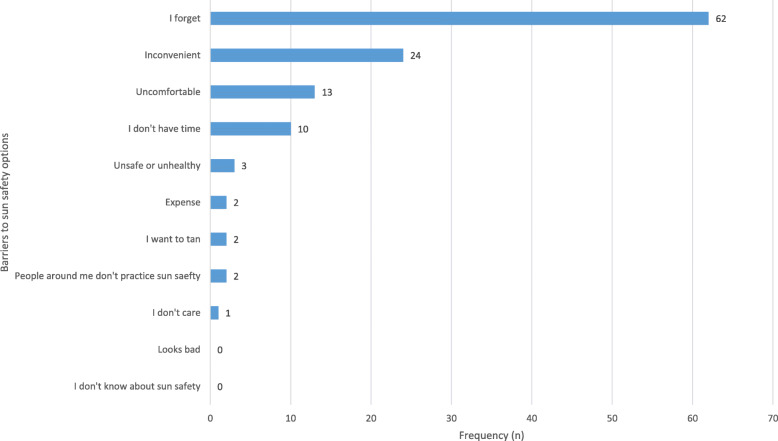
Frequency of Barriers to Sun Safety Options Amongst Farmers

Of the 144 participants, 89 (62.2%) identified one or more barriers present in comparison to 54 (37.8%) who did not identify any barriers to practicing sun safety. The most common barrier identified was “I forget” (43.4%, *n* = 62; Fig. 1). This was followed by “inconvenient” (16.8%, *n* = 24), “uncomfortable” (9.1%, *n* = 13), “I don’t have time” (7.0%, *n* = 10) and “unsafe or unhealthy” (2.1%, *n* = 3). No statistically significant association was found between barriers to sun safety and demographic variables (data not shown).

The association between sun safety practices and knowledge was explored. There was no significant correlation found between the two variables (*R =* − 0.051, *p* = 0.555). The association between the presence of barriers to sun safety and knowledge score was also examined. There was no significant difference (*p* = 0.321) between mean knowledge scores in people who reported barriers to sun safety (mean 8.3, SD 1.5) versus those that did not (mean 8.6, SD 1.2). The impact of gender on sun safety knowledge, practices and barriers was also examined (Table 5). No significant differences were found.

**Table 5:** Association between gender and participant knowledge score, sun safety practices score or barriers to sun safety amongst farmers

	Mean or frequency	SD or %	***T***-value or Chi-square value	df	***P***-value
**Sun safety knowledge score**					
Male	8.3	1.5	−1.261	134	0.209
Female	8.6	1.3			
**Sun safety practices score**					
Male	14.4	4.2	−1.216	141	0.226
Female	14.7	4.2			
**Barriers to sun safety present**					
Male	58	65.2	0.861	1	0.353
Female	31	57.4			

## Discussion

This study sought to characterise the current sun safety practices in a sample of farmers in Western NSW and explore their knowledge, attitudes and perceived barriers to sun safety on the farm. Overall, knowledge of sun safety was high and attitudes towards it were positive, though there was underestimation of the risk of developing skin cancer as only two-thirds of farmers reported that they were at higher risk of skin cancer when occupational exposure places all farmers at potentially higher risk of skin cancer than the general population. This finding was particularly surprising given that almost half of our participants reported having had at least one skin cancer removed previously as well as more than one third reporting they had 2 or more painful sunburns in the previous 12 months. The presence of barriers to implementing sun safety was significantly associated with reduced sun safety practices. The deficiencies in practices, knowledge, and attitudes identified in this study can be addressed with public health policy with the ultimate aim of reducing skin cancer prevalence and mortality in Australian farmers.

In this cohort from Western NSW, the presence of barriers to implementing sun safety practices was the biggest predictor of sun safety practices score, with those reporting barriers engaging in fewer sun safety practices. The most common barriers were forgetfulness, inconvenience, discomfort and time constraints. This is corroborated by other studies which reported similar barriers [20, 25]. Only one person identified “I don’t care” as a barrier in this study and a large majority affirmed sun protection was important to them. This is in contrast with other studies which have reported disregard for need of sun protection as an important barrier to engaging in sun safety behaviours [20]. Work conditions specific to farms have been hypothesised to contribute to barriers [20, 21, 25]. Further exploration of “discomfort” revealed that the texture of sunscreen with dust and dirt that accompanies farm work is undesirable, as is wearing long-sleeved trousers and shirts in the heat [20, 25]. A systematic review conducted by Smit-Kroner & Brumby found that some forms of sun protection such as avoiding sun during peak-times yielded greater barriers to farmers due to impracticality [11].

In order to improve sun safety practices, it is important to consider the practicality, comfort and convenience of sun protection measures. Since forgetfulness was the most common barrier identified in this study, some suggestions to encourage improvement would be advocacy of reminder systems including stickers or phone applications [25]. Further, perhaps the presence of sun safety protocols in the workplace, with endorsement from management, could help overcome these barriers. Multiple agencies, both governmental and non-governmental, are providing example policies and incentives such as tax-deductible personal protective equipment, to encourage the use of sun protection in the workplace [26, 27]. Recognition of skin cancer as an occupational disease in outdoor workers, such as farmers, as well as mandatory sun protection policies for outdoor workers have been associated with decreases in skin damage and cancer incidence [28, 29]. Indeed, only just over half of participants reported having a sun safety protocol at their workplace, so there is scope for improvement in this area. To examine if this finding was driven by a general lack of awareness of their workplace sun safety protocol amongst farm workers as compared to farm owners/managers, we explored the association between farm role type and reporting the presence of a sun safety protocol in the workplace. No significant association was found. Nonetheless, this issue could be explored in more depth in the current study region using qualitative research to permit a deeper probe into the specifics of work processes and the work environment on each farm and their impacts on sun safety practices. This would enable the identification of potential strategies that could be tested to enhance sun safety practices on the farm. There have been calls internationally for the establishment of health surveillance processes to monitor and assess occupational heat stress in outdoor workers, as this is predicted to increase with the ongoing effect of climate change across the globe [30, 31]; the impact of ozone depletion and changing UVR levels is another important environmental consideration. Alongside the direct implications of climate change on farming in Australia (e.g. drought and extreme weather events), Australian farmers will also be at risk of heat stress from these changing occupational conditions, making it important for ongoing research in this area to positively influence sun safety practices and other related occupational health and safety processes on the farm such as heat-stress mitigation.

Sun safety knowledge amongst participants was relatively high and sun safety attitudes were generally positive. This suggests that there is not a need for specific education on sun safety in this region, but rather more support with implementing/actioning knowledge and attitudes on the farm, as discussed above regarding barriers to sun safety. Certainly, another study reporting on sun protection knowledge of outdoor workers including farmers, telecom workers, and tradespeople, found that knowledge was relatively high, which was thought to reflect education from of a variety of sun safety campaigns [32]. However, they also found that knowledge did not directly translate into behaviours. Future research can seek to build this evidence base and evaluate strategies for overcoming barriers on the farm.

Only 60% of farmers reported a belief that they are at higher risk of developing skin cancer compared to the average population. This is similar to other studies reporting a rate of 40–50%, although Woods et al. reported a rate as high as 80.2% [19, 20, 33]. Higher annual household income was associated with increased perception of disease risk [19]. This study had a lower proportion of the cohort reporting annual household income greater than $100,000 (24.6%) compared to the New England, NSW cohort (45.3%) and may account for the difference in perception of disease risk [19]. The deficits in perceived risk of developing skin cancer are concerning considering there is well documented research showing that farmers are at increased risk [5]. Further, perception of disease risk is important as it may improve health behaviours resulting in positive health outcomes [5]. Health policy should aim to improve understanding of risk in populations who are a potentially higher risk, such as farmers, as a means to improve uptake of sun safety practices and thus reduce prevalence, morbidity and mortality associated with skin cancer.

To our knowledge, this is the first study to explore the practices, knowledge, attitudes and barriers to sun safety in farmers in Western NSW, although attention is starting to be turned to this issue in other rural areas of Australia. Despite the use of a convenience sample of participants, the events recruited from were well attended and attracted a wide sample of farmers from Western NSW. Comparison of the demographics of the participant sample with those reported for the farming workforce showed good consistency in terms of gender balance and mean age of the cohort suggesting applicability of the findings to the broader farming population. However, it should be acknowledged that the use of a convenience sample may have biased our sample towards those with more “positive” sun safety attitudes and behaviours. Indeed, this may explain why only one person reported “I don’t care” with regards to sun safety in this study as compared to other studies where this has been more commonly reported. If this is the case, then it may be that farmers in Western NSW have poorer sun safety habits than reported here, highlighting the need for a continued research focus on this area. Another key limitation of the study was the use of self-reported data. Level of education and participant knowledge may influence accuracy of reporting data such as skin cancer types and skin type according to the Fitzpatrick scale. Further, there was no distinction made between commercial and hobby farms. This could be important when considering which farms had sun safety protocols or not. It may be likely that those with a formal protocol are commercial enterprises and those without one are hobby farms.

Future research should involve multicentre studies with large sample sizes across Australia to determine whether these findings are replicable in farmers in other regional and remote areas. Involvement of health professionals in the assessment of skin type could help to increase accuracy. It would be useful to study the implementation of formal or informal sun safety protocols and the impact they have on sun safety practices to determine whether they reduce the instances of forgetting and help overcome other potential barriers as well. Differentiation between practices on commercial and hobby farmers is also a potential avenue for future research. Additional qualitative research would assist in better understanding the barriers to sun safety and allow participants themselves to describe how they could overcome these barriers.

## Conclusion

This study provides a snapshot into the current sun safety practices, knowledge, attitudes and perceived barriers in a sample of farmers working in Western NSW. Overall, there is good knowledge and considerable engagement. However, perceived barriers including forgetfulness prevent the optimal practice of sun safety. Future research is needed to examine perceived barriers to sun safety in more detail and explore how these can be overcome in this cohort to contribute to skin cancer prevention.

## Data Availability

All data are available from the corresponding author upon reasonable request.

## Abbreviations

NSW: New South Wales
SPF: Sun Protection Factor
UVR: Ultraviolet Radiation
WNSW: Western New South Wales
